# Branched and Fenestrated Aortic Endovascular Grafts

**DOI:** 10.14797/mdcvj.1200

**Published:** 2023-03-07

**Authors:** Aidan D. Atkins, Marvin D. Atkins

**Affiliations:** 1 Texas A&M University Department of Biomedical Engineering, College Station, Texas, US; 2 Houston Methodist DeBakey Cardiovascular Surgery Associates, Houston, Texas, US

**Keywords:** branched endograft, fenestrated endograft, physician modified endograft, thoracic aortic aneurysm, thoracoabdominal aortic aneurysm, complex abdominal aortic aneurysm

## Abstract

Endovascular repair of abdominal and descending thoracic aortic aneurysms has become the standard of care due to improvements in morbidity and mortality compared to open surgical repair. Late durability, however, remains an issue because persistent endoleaks can lead to continued aneurysm expansion and eventual rupture, sometimes years following the original repair. Branched, fenestrated, and physician-modified endografts in the thoracic arch and thoracoabdominal aorta have extended the seal zone in order to mitigate the risks of proximal and distal endoleaks. This review summarizes the current state of branched, fenestrated, and physician-modified endografts used in complex aortic pathologies.

## Introduction

Endovascular stent grafts have revolutionized the management of abdominal and thoracic aortic aneurysms since their original descriptions by Parodi in 1990 and Dake in 1992.^1,2^ Since that time, endovascular aortic aneurysm repair (EVAR) and thoracic endovascular aortic repair (TEVAR) have become the first-line treatment options for abdominal and thoracic aneurysm repair due to their many benefits, including decreased blood loss, operative time, length of stay, morbidity, and mortality compared with conventional open surgical repair. Successful EVAR and TEVAR are predicated upon landing the devices in the nonaneurysmal, parallel aorta to create a seal zone both proximally and distally. In up to 40% of patients, endovascular repair is limited by anatomical constraints such as inadequate neck length, aortic angulation, or proximal extension of the aneurysm to include one or more visceral or brachiocephalic vessels.

EVAR’s primary challenge is a difficult proximal sealing and fixation zone, particularly in patients who have a short or angulated infrarenal aortic neck. Type I endoleaks, due to an unfavorable proximal sealing zone, can have dire consequences in the long-term, such as continuing aortic dilatation in the paravisceral segment of the aorta leading to endograft failure and ultimately rupture. Similar issues also occur in the thoracic aorta, where the proximal or distal extent of the aneurysm do not leave enough normal parallel aorta for sealing. Coverage of aortic branches has been used successfully in the thoracic aorta, with coverage of the left subclavian artery and even the celiac artery owing to the robust collateral circulation around each. Such coverage is not typically feasible in the abdominal aorta.

Extending the seal zone to normal paravisceral or supraceliac aorta with incorporation of branch vessels using fenestrations, scallops, or directional branches has been used successfully. A fenestration is a hole in the graft, typically 6 or 8 mm in diameter and reinforced with suture and radiopaque markers, that is custom located corresponding to the patients’ anatomy. The fenestration is typically bridged with a covered stent graft, and the proximal end of the bridging stent is flared inside of the main body endograft. Scallops are U-shaped cut-outs in the proximal portion of the endograft to incorporate the most proximal branch vessel, typically the superior mesenteric artery (SMA) or celiac artery. Branches are small sections of graft that are sewn to the main body endograft that are typically downward facing. These side branches allow for increased seal zone combined with the bridging stents; however, they necessitate a more proximal deployment of the main body endograft, increasing the risk for paraplegia.

Browne et al. reported the first fenestrated aortic repair in 1999 to treat a juxtarenal aneurysm with a single fenestration for a renal artery.^3^ Anderson reported the first use of a fenestrated endograft that incorporated both renal arteries and the SMA based upon the Cook Zenith EVAR platform.^4^ In patients without a seal zone in the infrarenal aorta, custom fenestrated endovascular stent grafts have been available in US-based clinical trials since 2006. These devices were approved for short neck (4-15 mm) abdominal aortic aneurysms with a seal zone created within the visceral segment. Since that time, only the Cook ZFEN Fenestrated endograft has been commercially approved in the US (in 2012). Multiple devices from various manufacturers are currently undergoing clinical trials, with several more on the horizon. Due to the lack of commercially available branched or fenestrated devices, many sites have used physician-modified endografts, where an endograft is deployed on the back table and the grafts are modified with either fenestrations or branches and reloaded back into the delivery sheath for implantation. Another option described in detail is the use of a physician-sponsored investigational device exemption. This program allows US physicians to apply to the FDA for a surgeon-specific clinical trial protocol to test newer endografts and/or physician-modified endografts.^5^

The current literature suggests that as with EVAR, the morbidity and mortality resulting from a fenestrated or branched endograft procedure is at least comparable in the short term to open surgical repair.^4^ Most fenestrated grafts, however, have been implanted in those deemed high risk for open repair, so there are no direct comparisons between the two treatment strategies. The long-term durability of such grafts, especially physician-modified endografts, is unknown. Reinterventions for device migration, component separation, or branch vessel stenosis or occlusion are not uncommon. As with EVAR, most patients would still choose a minimally invasive option for aneurysm repair even if it means more frequent surveillance and reintervention. This article provides an update on the current status of branch and fenestrated endografts for use in the abdominal, thoracoabdominal, and thoracic aorta.

## Physician-Sponsored Investigational Device Exemption

Aside from industry sponsored clinical trials, another means of evaluating new endovascular devices or physician-modified devices is a physician-sponsored investigational device exemption (PS-IDE). A PS-IDE allows a device not approved by the FDA to be studied similarly to a clinical trial. With this designation, the physician is both the investigator and the sponsor, assuming a greater responsibility than he or she would in an industry-sponsored IDE. The IDE process includes creating a research study protocol and case reporting forms, and submitting an IDE request to the FDA. A study coordinator and monitor are required to ensure the informed consent process, capture all study data per the protocol, and report adverse event data to the institutional review board (IRB) and the FDA. Yearly study reports to the IRB and FDA are standard, as are data audits and case reporting, since the purpose of the IDE is to capture data similar to a clinical trial. By enrolling patients and capturing data as part of an IDE, the procedures are typically eligible for coverage by Medicare with reimbursement.

The process of obtaining a PS-IDE can be daunting, but multiple clinical sites around the US have navigated the process and can offer complex endovascular AAA repair to patients. Several sites have joined together as part of the US Fenestrated and Branched Aortic Research Consortium. Single center reports have shown excellent results compared to open surgical repair with regard to operative mortality, freedom from device-related adverse events, late rupture or migration. The limitation of these reports is typically the small sample size. Ten of the busiest PS-IDE sites, which have obtained a PS-IDE to evaluate various Cook medical devices and physician-modified endografts, have pooled their data to provide the largest dataset of branched/fenestrated procedures in the world. This collaboration aims to answer complex questions regarding the burgeoning field of branch and fenestrated abdominal aortic aneurysm repair.

## Abdominal Fenestrated Endografts

The following sections describe the current branched or fenestrated endovascular devices that are commercially available, on trial in the US, or are part of a current PS-IDE. Physician-modified endografts and parallel grafts (including chimneys, snorkels, and periscopes) are considered elsewhere in this journal.

### Cook ZFEN Device

At the present time, the Cook Zenith ZFEN device is the only commercially approved fenestrated device in the US. In 2001, Anderson and Hartley reported the first use of a 3-vessel fenestrated graft in Australia based on the Cook Zenith platform.^6^ The Cook Zenith ZFEN device is indicated for juxtarenal AAA with at least a 4-mm infrarenal neck. Since commercial approval, it has been used in patients with little or no aortic neck with reasonable success. The Cook Zenith Fenestrated AAA Endovascular graft was commercially approved by the FDA in 2012 and has been commercially available outside the US since 2002. Since 2019, there have been approximately 8,500 implants of the Cook ZFEN proximal device component in the US.

**Figure 1:**
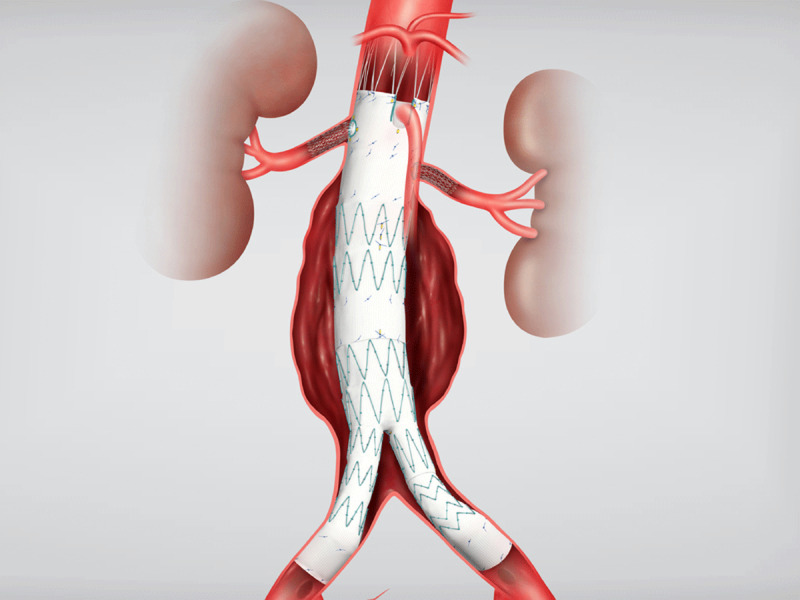
Cook Fenestrated ZFEN device. Courtesy of Cook Medical.

The device is custom built for each individual patient’s anatomy. The device typically consists of two fenestrations for the renal arteries and either a fenestration or scallop for the SMA (Figure 1). The turnaround time for construction is between 4 to 6 weeks, precluding its use in urgent or emergent situations.

In 2019, Cook Medical published their annual clinical update on the ZFEN device.^4^ This 5-year long-term study consisted of 88 patients, including 67 patients enrolled premarket and 21 enrolled following approval of the device. Freedom from AAA-related mortality at 60 months was 97.5%. There was one death on postoperative day 2 secondary to bowel ischemia and a second death of unclear etiology at 2 years that was conservatively counted as AAA related. No death was related to device failure or aneurysm rupture. Freedom from all-cause mortality was 87.9% at 5 years. There was one rupture in the setting of a type 3 endoleak and component separation (which was salvaged endovascularly) occurring at the 5-year follow-up. There were no conversions to open repair or graft explantations. One patient developed acute renal failure requiring dialysis within 30 days. Three patients developed bowel ischemia, with two resolving following intravenous fluids and antibiotics. The third was the above patient who died on postoperative day 2. Main body device stent fracture occurred in two patients and limb separation in one. Four patients required secondary reinterventions due to stenosis or occlusion of renal artery stent grafts. Aneurysm sac size decreased in 76.3% of patients at 5 years, was stable in 18.4%, and increased in 5.3%. Graft migration (defined as > 5 mm with renal stent compression) occurred in one patient and required reintervention. Three patients experienced type 1 proximal endoleak while two had distal endoleak, and all required reintervention. Six patients had type 2 endoleaks due to sac enlargement requiring intervention via coil embolization.

The above results are favorable compared with the open treatment of juxtarenal AAA and in a patient population deemed high risk for open surgical repair. The limiting factor for use of the ZFEN device is unusual patient anatomy such as small renal arteries (< 4 mm in diameter), severe aortic angulation, or unusual spacing between the SMA and the renal vessels precluding construction of the device. As the 5-year ZFEN follow-up would suggest, late reinterventions are not uncommon and highlight the need for lifelong surveillance.

### Cook P-Branch

**Figure 2:**
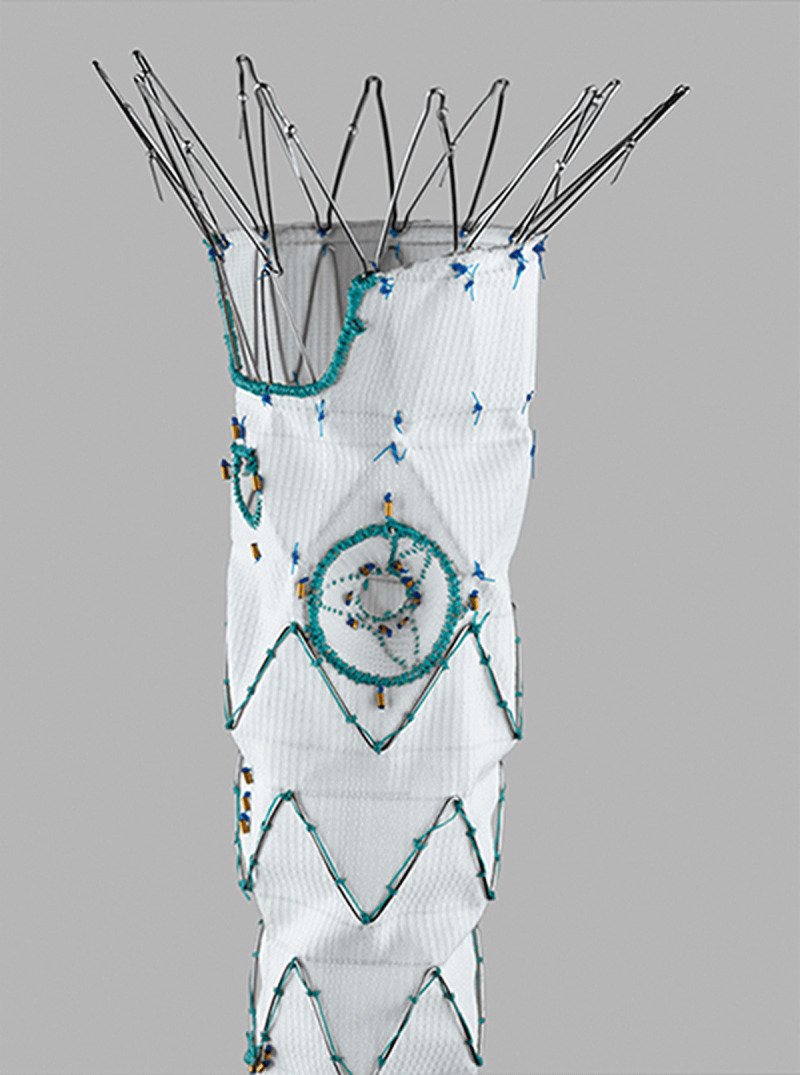
Cook p-Branch device with hinged, adaptive renal fenestration. Courtesy of Cook Medical.

The Cook p-Branch is an “off-the-shelf” design that incorporates three fenestrations and a scallop for the celiac artery. It is designed for aneurysms that start below the SMA origin (Figure 2). The US pivotal p-Branch study has required an infra-SMA neck length of 15 mm. The p-Branch device is a modular system that is 26 mm by 36 mm in diameter. The primary section has a supraceliac uncovered barbed stent followed by nitinol z-stents with one scallop for the celiac artery, one fixed 8-mm strut-free fenestration for the SMA, and two hinged adaptive renal artery fenestrations. These adaptive conical fenestrations have an outer diameter measuring 15 mm at the level of the aortic graft and 6 mm in diameter distally. The small conical branch allows for greater mobility of the stent due to variation in renal anatomy. There are two available renal configurations, one with the renals at the same level and the other with the left renal 4 mm below the right renal artery. The bridging stent used in the trial was the Atrium icast covered stent. In one single-center IDE study, the p-branch device would be applicable to between 60% and 70% of patients presenting with juxtarenal and suprarenal aneurysms.^7,8^ Utilizing the strict criteria in the US p-Branch pivotal trial, only a third of patients would meet such strict anatomic criteria.

Based on the European experience with the p-Branch device, the adaptive fenestrations for the renal arteries have shown decreased target vessel patency during follow-up.^9^ The mechanism appears to be increased strain on the mating stents when the fenestrations are offset and the fenestration is less stable.

The off-the-shelf p-Branch device solves the issue of device availability. The proximal p-Branch component, which has two configurations and five diameters, can be kept on hand in limited numbers. The uniformly designed distal bifurcated body is available in four lengths. The procedure is completed with the standard iliac extension limbs used for an infrarenal Zenith device. Thus, a total of 14 devices in stock will cover most anatomies within the instructions for use and is ideal for urgent or emergent use.

The Zenith p-Branch Pivotal Study began enrollment in August 2015 and included 80 patients.^10^ Having completed enrollment, the 12-month results were recently submitted to the FDA for audit. Publication of the results will occur in 2023; pending a positive outcome, the device should be commercially available between 2023 and 2024.

## Thoracoabdominal Fenestrated Endografts

### Gore TAMBE

**Figure 3:**
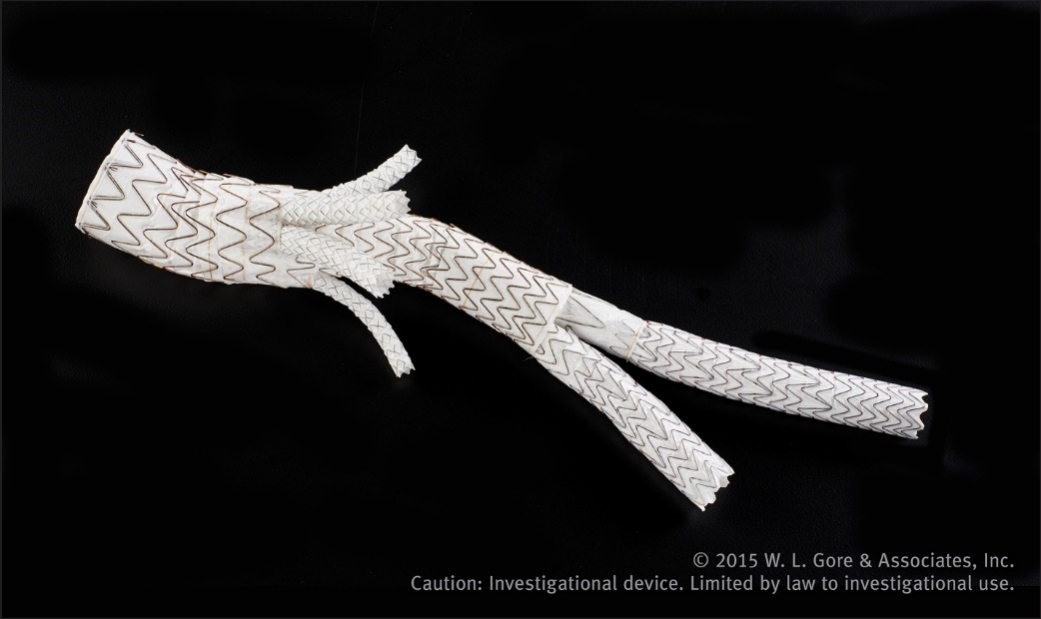
Gore TAMBE thoracoabdominal device. GORE^®^ EXCLUDER Thoracoabdominal Branch Endoprosthesis © 2023.

The Gore EXCLUDER Thoracoabdominal Branch Endoprosthesis (TAMBE) device is an off-the-shelf four-branch endograft indicated for the treatment of pararenal and type 4 thoracoabdominal aortic aneurysms (TAAA) (Figure 3). The device is currently being studied in the US and Europe in two independent study arms—the primary using solely the TAMBE device to treat pararenal and type 4 TAAA and the secondary using the TAMBE device in addition to proximal conformable GORE TAG thoracic endograft devices placed proximally for type 1,2, and 3 TAAA.

Cambiaghi et al. examined the anatomical feasibility of the Gore TAMBE device on all patients presenting at a single European institution with pararenal and TAAA between 2007 and 2017.^11^ This included patients undergoing open and endovascular approaches in either elective or emergent settings. The anatomical inclusion/exclusion criteria for the US Pivotal trial were used. In those presenting with pararenal aneurysms and type 4 TAAA, 49% of cases could have been treated with the TAMBE device alone. Access feasibility was 85%, aortic feasibility was 74% and visceral vessel feasibility was 72%. In those presenting with type 1, 2, and 3 TAAA, only 23% of cases could have been treated with the TAMBE device with proximal C Tag extensions. Access feasibility was 79%, aortic feasibility was 48% and visceral vessel feasibility was 63%. The low aortic feasibility rate in type 1, 2, and 3 TAAA was felt to be due to size differences between the thoracic aorta and the visceral segment and lack of a dedicated tapered thoracic component.

The Gore TAMBE device involves four down-going branches for the celiac, SMA and renal arteries. Deployment involves placement of a 12F sheath via a left axillary artery cut down. A wire is passed from the left axillary sheath and snared through a 22F sheath via the femoral artery. The wire is externalized and a proprietary multichannel catheter is advanced over the wire. Four .014 wires are advanced through the multichannel catheter at the axillary sheath and externalized and advanced through portals in the TAMBE graft to precannulate the side branches. The multichannel catheter prevents wire wrap when advancing the device. The TAMBE device is then introduced to the paravisceral segment and partially deployed. Each .014 wire is then used with a catheter from above and brought into each branch and used to cannulate the corresponding vessel. This wire is then exchanged for a 0.035 Rosen wire. Once all four branch vessels have been cannulated, the TAMBE device is fully deployed. Branching VBX stents are then brought down through a 7F sheath from above and deployed from the TAMBE branch into the branch vessels. The TAMBE device is ballooned above the branches at the proximal seal zone. A bifurcated bridging piece is then deployed into the TAMBE device and extended down into each Iliac artery with Gore Excluder Iliac Branch Endoprosthesis.

As of March 2021, this prospective multicenter nonrandomized study involving 45 sites across the US and Europe had enrolled 106 and 23 patients for the primary and secondary study arms, respectively. The primary study arm is complete, with subjects continuing in follow-up. The trial is currently enrolling in the US and Europe and initial results are forthcoming. The device has received Breakthrough Device designation from the FDA.

### Cook T-Branch

**Figure 4:**
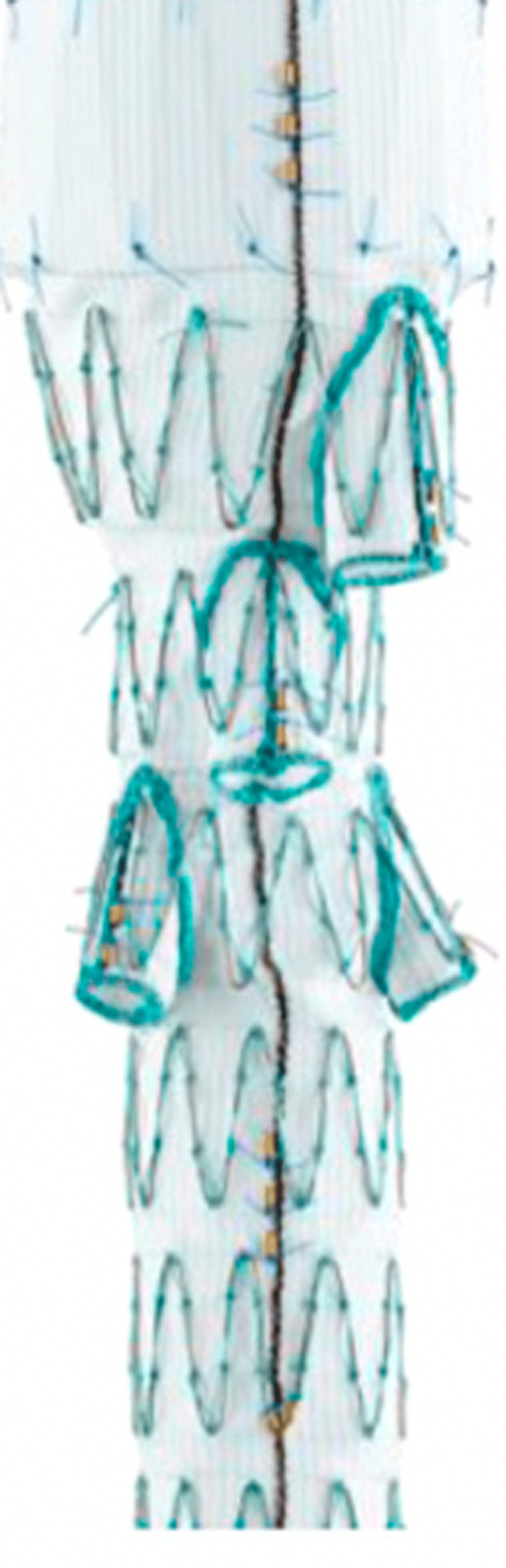
Cook t-Branch thoracoabdominal device. Courtesy of Cook Medical.

The Cook Zenith t-Branch graft is a single configuration off-the-shelf solution for endovascular repair of TAAAs (Figure 4). The graft comes in one size, 34 mm proximally and 18 mm distally, with four downward branches that are accessed from above via the left subclavian artery. The proximal graft contains barbs for additional device fixation. The graft connects to the celiac, superior mesenteric, and renal arteries by way of self-expanding covered vascular bridging stents. The graft includes four gold radiopaque markers to facilitate fluoroscopic visualization and ensure orientation for deployment.The device is currently involved in three ongoing PS-IDE trials in the US.

There is significant worldwide experience with the t-Branch device. Bosiers et al. reported the results from three European centers including 80 patients undergoing endovascular repair of TAAA, with 72% performed electively, 7.8% symptomatic, and 19.5% presenting with a contained rupture.^12^ The t-Branch device was successfully deployed in 79/80 patients. Although one patient died within 30 days and seven patients (8.8%) after 1 year, none were related to aneurysm rupture or explantation.

### Medtronic Valiant TAAA

**Figure 5:**
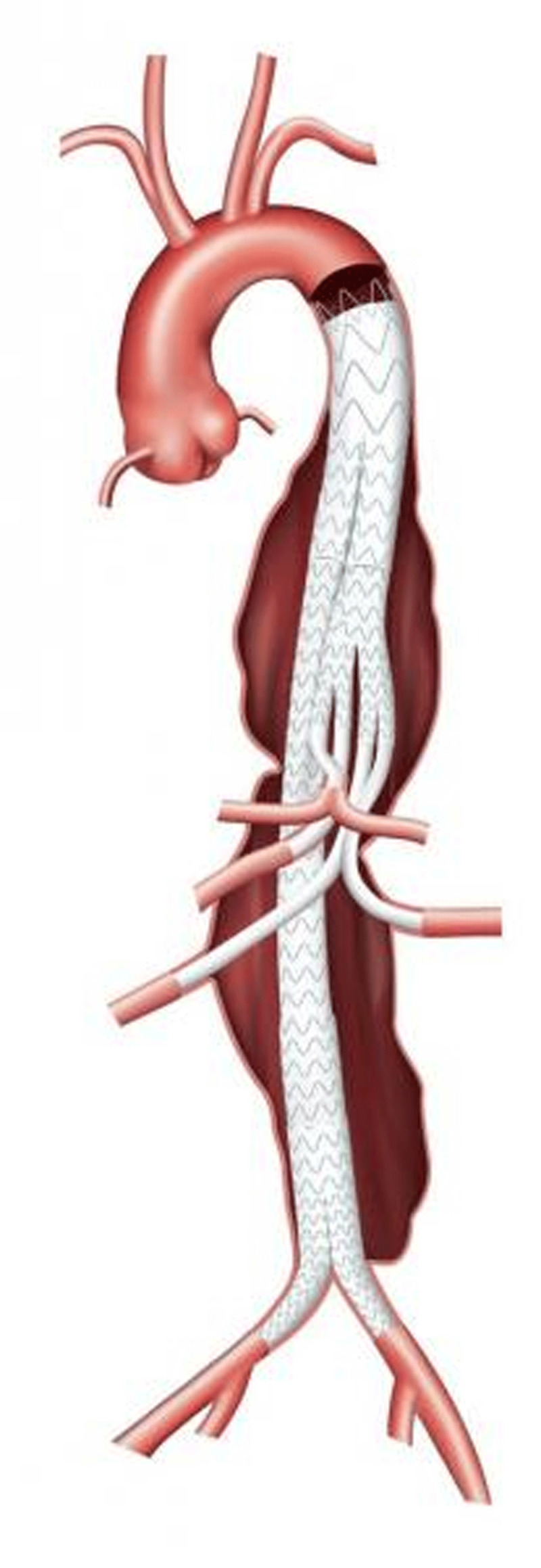
Medtronic Valiant thoracoabdominal aortic aneurysm device. Used with permission.

The Medtronic Valiant TAAA device is an off-the-shelf treatment for TAAA (Figure 5). The device is designed to deploy proximally in the descending thoracic aorta. The system contains two investigational devices: the thoracic bifurcation and the visceral manifold. The thoracic bifurcation is deployed in the proximal thoracic aorta and provides the proximal seal. The two limbs of the thoracic bifurcation allow for continued aortic flow while deploying the visceral segment. The visceral manifold is deployed within the larger 20-mm limb of the thoracic bifurcation to set the stage for the visceral debranching. Each individual visceral branch is sequentially cannulated from above via a sheath in the left subclavian artery. The branches of the visceral manifold extend to the visceral vessel via covered bridging stents and provide distal seal of the manifold.

As of October 2019, there were five US sites with PS-IDE trials examining the Medtronic Valiant TAAA device, and it had received Breakthrough Device Designation from the FDA. In 2001, this group reported their analysis of 139 patients who underwent treatment with the Medtronic Valiant thoracoabdominal stent graft system.^13^ The majority of patients (94.2%) underwent a single procedure rather than staged delivery of the branch grafts. Thirty-day, 1-year, and 2-year all-cause mortality rates were 5.8%, 25.2%, and 32.4%, respectively. Paraplegia was noted in 5 patients (3.6%) with 40.3% of cases in Crawford I-III TAAA. Primary and secondary vessel patency rates were 96.2% and 97.5% for all vessels and 95.4% and 96.9% for renal arteries at a median follow-up of 26.9 months. Acute kidney injury (AKI) was reported in 22 patients (15.8%), with 3 requiring permanent dialysis. Five more patients developed worsening renal function and subsequent dialysis through the 2-year study period. In this high-risk population, the Medtronic Valiant TAAA device was thought to be a reasonable solution. The study highlighted the somewhat significant rates of worsening AKI and need for permanent hemodialysis in this patient population.

### Cook ZFen

The commercially available Cook ZFEN device is only approved for a maximum of three custom fenestrations and only a single wide 10-mm scallop. Depending on the device configuration, one of the stent struts may cross the fenestration openings. This can significantly impair the ability to deploy a bridging stent and require back table modification of the strut. It is estimated that two-thirds of complex juxtarenal and pararenal aneurysms are not covered by the ZFEN design.^1,2^ The Zenith Fenestrated + Endovascular graft (ZFEN +) is an investigational stent graft in preclinical phase that improves upon the current ZFEN design. ZFEN + device is designed for pararenal and type 4 TAAA with a seal created in the supraceliac aorta. The device allows for up to five small or large reinforced fenestrations and incorporates preloaded catheters to facilitate the procedure. This device design has been available outside the US and in select US centers via PS-IDE protocols. The ZFEN + pivotal trial is a prospective international multicenter nonrandomized clinical study in presubmission phase pending IDE submission to and approval by the FDA. It is expected that the ZFEN + device will be applicable to most patients with complex AAAs.

### Terumo Aortic Custom Fenestrated Anaconda

The Terumo Anaconda EVAR device is currently undergoing phase 2 testing in the US for treatment of infrarenal AAA. The custom fenestrated Anaconda device, based upon the same platform, has been used outside of the US for several years for complex juxtarenal and pararenal AAAs. The device can be constructed with up to four fenestrations for the visceral vessels. Data from a UK multicenter study of the fenestrated Anaconda device in 101 patients showed 97.6% target vessel patency at 1 year.^14^ At 1 year, 75.8% of patients exhibited aneurysm sac regression and 23.1% showed stable aneurysm sac size. Freedom from reintervention at 1 year was 91%. There are currently two US sites enrolling in PS-IDE trials to examine this graft.

## Thoracic Branched Endografts

### Gore Thoracic Branched Endograft Device

**Figure 6:**
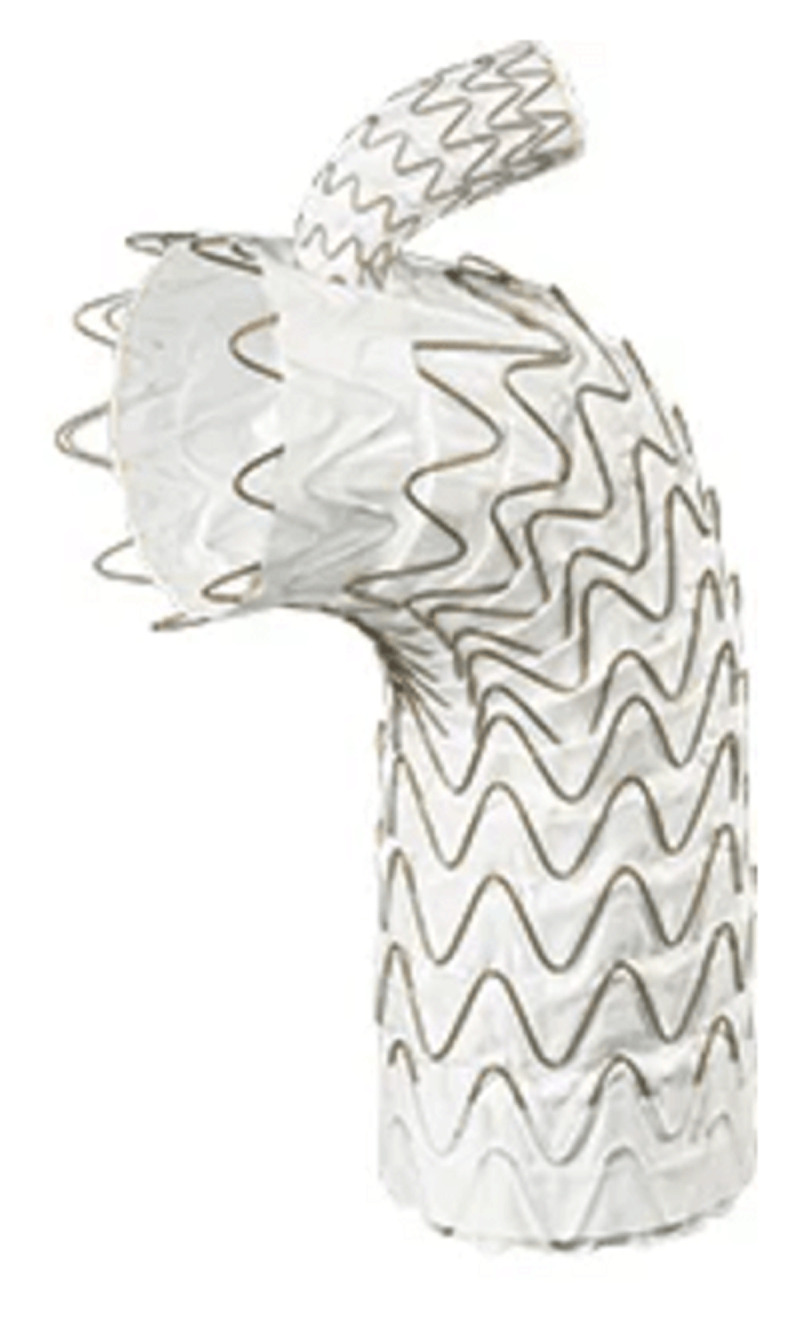
Gore thoracic branch endograft device. GORE thoracic branch endograft © 2023.

The Gore Thoracic Branched Endograft (TBE) device is the only commercially approved branched thoracic stent graft available in the US (Figure 6). This was approved in May 2022 for use within the left subclavian artery (ie, zone 2). The TBE device incorporates a single internal retrograde branch for arch vessel perfusion. The 3-year midterm results of the early feasibility trial were recently published, showing favorable patency and durability with low rates of graft related complications.^15^ This cohort consisted of 40 patients (31 zone 2 and nine in zone 0/1). There were no device migrations, stent fractures or aortic ruptures. Freedom from death at 1 and 3 years was 90% and 84%, respectively. A second arm of the study involves deployment of the side branch in zone 0/1. This involves deployment in either the innominate or left common carotid artery with extra-anatomic bypass of the left common carotid or left subclavian artery.

Results from the US Pivotal trial for the Gore TBE device were recently published and resulted in the device’s commercial approval.^16^ This study involved 34 investigational sites and reported the 1-year results of 84 patients who underwent treatment of descending thoracic aneurysms with graft deployment in zone 2 and a side branch into the left subclavian artery. Technical success was achieved in 92% of patients (77/84). There were no aneurysm-related mortalities at 12 months. There was a single case of paraparesis (which completely resolved by 1 year) and a single case of paraplegia that partially resolved. Three patients experienced a procedure-related stroke. One case of type 3 endoleak required reintervention. The device is also being studied in three other cohorts, namely traumatic transection, dissection, and other lesions (penetrating ulcer, IMH) and published results in those cohorts are pending.

### Medtronic Valiant Navion LSA Device

Medtronic endovascular had previously developed and studied a left subclavian branch device based on its Valiant thoracic stent graft platform. The Mona-LSA stent graft had previously undergone early feasibility testing, and results were published in 2015.^17^ The device was then changed to include the Navion thoracic stent graft platform, which was recalled by the FDA and taken off the market in February 2021 due to stent graft fractures and endoleak concerns. The future status of the Mona LSA branched thoracic stent graft device is unknown.

### Terumo Relay Branch Device

In 2017, Terumo Aortic launched the early feasibility study of their Relay Branch thoracic graft platform in the US. The Relay Branch system is intended for the treatment of thoracic aortic arch pathologies, including aneurysm, dissection, and intramural hematoma requiring coverage of the innominate and left common carotid arteries. The proximal end of the device is intended to land in zone 0 in the ascending aorta. There is a large cannulation fenestration leading to two antegrade internal tunnels. The right and left common carotid arteries in the neck are accessed to deliver the antegrade branches sequentially into the innominate and left common carotid arteries.

Worldwide experience with the dual-branch Relay device has been favorable. Tan et al. reported on multicenter data from Europe in 148 patients who underwent treatment of arch pathology with the Terumo Relay Branch device from 2019 to 2022.^18^ Technical success was achieved in 99.3% of cases. There was one mortality following the procedure but was not thought to be device or procedure related. Six patients (4.1%) developed a disabling stroke and eight (5.4%) suffered a nondisabling stroke during the 2 year follow-up period. Reintervention was required in 16.3% by 24 months and branch vessel patency was 80% (118/147) at 2 years. This experience highlights the major issue of endovascular therapy in the aortic arch, the risk of branch vessel occlusion and stroke.

### Cook Arch Branch

Cook endovascular has designed an inner branch arch endovascular graft. The custom graft seals in the ascending aorta and has up to three proximal internal sealing stents with active fixation barbs on the most proximal sealing stent. A retrospective multicenter analysis of the first 38 patients treated with this device was published in 2014.^19^ This early feasibility data included an early learning curve for experienced practitioners who have used the device in this high-risk patient cohort. Thirty-day mortality was 30% in the first 10 patients and 7.1% in the subsequent 28 patients, which confirms the significant learning curve with patient selection and procedural experience. This early feasibility study demonstrated reasonable results for total endovascular arch repair in high-risk patients.

Haulon et al. recently reported a systematic review on customized (branched and fenestrated) and noncustomized (parallel graft or surgeon-modified fenestrated TEVAR) device techniques for endovascular repair of the aortic arch.^20^ Their review of the literature from 2000 to 2022 included 30 studies and 2,135 patients. Pathologies included aortic dissection (48%) and aneurysm (46.9%). Custom designed fenestrated and branched custom devices had a technical success rate of 98.3% and 98.7%, respectively; 30-day mortality was 3.8% and 5.4%, and stroke rates were 12.3% and 11%. During 2-year follow-up, reinterventions were required in approximately 10% of patients.

The use of noncustomized device techniques with parallel grafts was associated with a technical success of only 76.4%. Physician-modified fenestrated TEVAR had a technical success rate of 91.6%. Mortality rates in the two noncustom techniques were 4.4% and 2.3%. Stroke rates were 4.3% and 3.5%, respectively. This review concluded that endovascular arch repair was associated with acceptable technical success and 30-day mortality in cohorts of high-risk patients. Cerebrovascular event rates averaged approximately 10% and highlighted the Achilles heel of endovascular arch repair.

### Physician-Modified Endografts

The role of back table thoracic aortic endograft modifications for use in the abdominal aorta, much less the aortic arch, remains largely unclear. The Society for Vascular Surgery (SVS) issued an advisory statement in 2013 regarding physician-modified devices and noted that both local IRB and FDA IDE approvals should be obtained prior to use of a physician-modified endograft device. In centers with a PS-IDE, physician-modified endografts are included in their reporting to the FDA. Current evidence would suggest that the results of physician-modified endografts in both the arch and thoracoabdominal aorta are reasonable in high-risk patients without other options. Such cases should be collected in a PS-IDE, and informed consent is paramount when counseling patients beforehand about the unknowns associated with modifying an endograft.

## Conclusion

Endovascular treatment of complex thoracic, thoracoabdominal, and abdominal pathologies continues to gain popularity as a valuable alternative to open surgery, especially in patients unfit for open repair. Endovascular stent graft repair of complex aortic anatomy appears to match, at least in the short-term, the outcomes of open repair with regard to mortality and morbidity. Issues of perioperative stroke continue to plague complex thoracic arch endovascular repair. Late branch vessel patency is a significant issue in the visceral segment of the aorta, especially with regard to the renal arteries and renal function. Continued improvement in endovascular design and deployment techniques will hopefully help to mitigate the above issues. The future of endovascular therapy in complex aortic disease is still in its early history and will continue to have an increasing role for patients.

## Key Points

- Custom fenestrated and branched endografts play an increasing role in the management of complex abdominal and thoracic aortic pathologies.
- Early results of such technologies show at least similar results in morbidity and mortality compared with open repair, although a direct comparison cannot be made as such technologies are typically applied to patients considered high risk for open repair.
- Late branch endograft failure, especially in the renal arteries, is a significant issue mandating continued surveillance.
- Acute periprocedural stroke is the Achilles heel of endovascular intervention in the aortic arch.
